# The effect of low and moderate intensity aerobic exercises on sleep quality in men older adults

**DOI:** 10.12669/pjms.302.4386

**Published:** 2014

**Authors:** Ahmad Ali Akbari Kamrani, Amir Shams, Parvaneh Shamsipour Dehkordi, Robabeh Mohajeri

**Affiliations:** 1 Ahmad Ali Akbari Kamrani, Iranian Research Center on Ageing, University of Social Welfare & Rehabilitation Sciences, Tehran, Iran.; 2 Amir Shams, Department of Physical Education and Sport Sciences, Shahid Beheshti University, Tehran, Iran.; 3 Parvaneh Shamsipour Dehkordi, Department of Physical Education and Sport Sciences, Shahid Beheshti University, Tehran, Iran.; 4 Robabeh Mohajeri, Department of Physical Education and Sport Sciences, Science and Research Branch, Islamic Azad University, Tehran, Iran.

**Keywords:** Adults, Aerobic Exercises, Older, Sleep Quality

## Abstract

***Objective:*** Sleep is an active and complex rhythmic state that may be affected by the aging process. The purpose of present research was to investigate the effect of low and moderate intensity aerobic exercises on sleep quality in older adults.

***Methods:*** The research method is quasi-experimental with pre-test and post-test design. The statistical sample included 45 volunteer elderly men with age range of 60-70 years-old that divided randomly in two experimental groups (aerobic exercise with low and moderate intensity) and one control group. In each group selected 15 older adults based on inclusion and exclusion criteria (such as, without sleep apnea, not smoking, and no taking hypnotic drugs). First, all subjects were evaluated by a doctor to confirm their physical and mental health. Also, the maximum heart rate (MaxHR) of subjects was obtained by subtracting one's age from 220. Furthermore, based on aerobic exercise type (40-50% MaxHR for low intensity group and 60-70% MaxHR for moderate intensity group) the target MaxHR was calculated for each subject. The exercise protocol consisted of 8 weeks aerobic exercises (2 sessions in per-week) based on Rockport one-mile walking/running test and the control group continued their daily activities. All subjects in per-test and post-test stages completed the Petersburg Sleep Quality Index (PSQI).

***Results:*** In pre-test stage, results showed that there were no significant differences between control and experimental groups in sleep quality and its components (P>0.05). On the other hand, results in post-test stage showed that there were significant differences between control and experimental groups in these variables (P<0.05). Also, the Tukey Post Hoc showed that the moderate intensity group scores in total sleep quality and its components were better than other groups (P<0.05). Finally, the low intensity group scores in total sleep quality and its components were better than control group (P<0.05).

***Conclusion:*** Generally, the present research showed that the aerobic exercises with moderate intensity (60-70% MaxHR) have a positive and significant effect on sleep quality and its components. Thus, based on these findings, the aerobic exercises with moderate intensity is a useful to improve the sleep quality and its components among community older adults were recommended.

## INTRODUCTION

Sleep is one of the vital aspects for overall health, especially in older adults. Sleep disorders and sleep disturbance are highly problems that reported by 39–75% of older adults.^1^^-^^3^ In the United States, the epidemiologic studies reported that almost half of the older adults have difficulty in sleep onset and maintenance.^3^^,^^4^ The most common sleep complaint in older adults is insomnia.^5^ Martin et al. reported that the direct and indirect costs of insomnia have been estimated at over $100 billion per year in the United States.^6^ Studies specific on the older adults indicated that symptoms of insomnia and sleep disturbances are associated with daytime dysfunction, poorer cognitive function and quality of life, depression, activity limitations, fatigue, emotional distress, increased risk of falls and increased incidence of cardiovascular morbidity and mortality.^3^^,^^5^^,^^7^ Furthermore, several studies have showed that older adults spend a smaller percentage of time in rapid eye movement (REM) sleep and a higher percentage of time in light sleep (stages 1 and 2 sleep).^8^^,^^9^

One method to improve and increase sleep quality is to take medication, but this method has side-effects (such as daytime residual effects, tolerance, dependence, and rebound insomnia).^10^ Thus, the use of non-medical or non-pharmacological methods appears to be necessary in older adults.

Based on the results of previous studies, one of the useful and without side-effects methods is physical activity. The maintenance of high physical function is one of the key factors for successful aging.^11^ Staying physically and mentally active can not only delay the development of some chronic illnesses and disabilities in older adults, but also improve sleep quality.^11^^,^^12^

In well-controlled laboratory settings, King and colleagues reported that older adults with moderate sleep complaints can improve and increase sleep quality by moderate-intensity exercise program.^13^^,^^14^ Also, previous studies have reported that Tai Chi or yoga activities may improve sleep quality.^15^^,^^16^ Although Tai Chi and yoga have been found beneficial in older adults, their complexity makes them difficult for older adults to learn.^5^ On the other hand, laboratory setting cannot provide for all individuals. Therefore, using the aerobic exercise in field setting seem to be necessary in older adults. Unfortunately, no study has examined the aerobic exercise effects on sleep quality in field setting, so that all older individuals can use its results and exercise trainings. Thus, the purpose of this study was to examine the effects of low and moderate intensity aerobic exercises on sleep quality in older adults.

## METHODS

**Fig.1:**
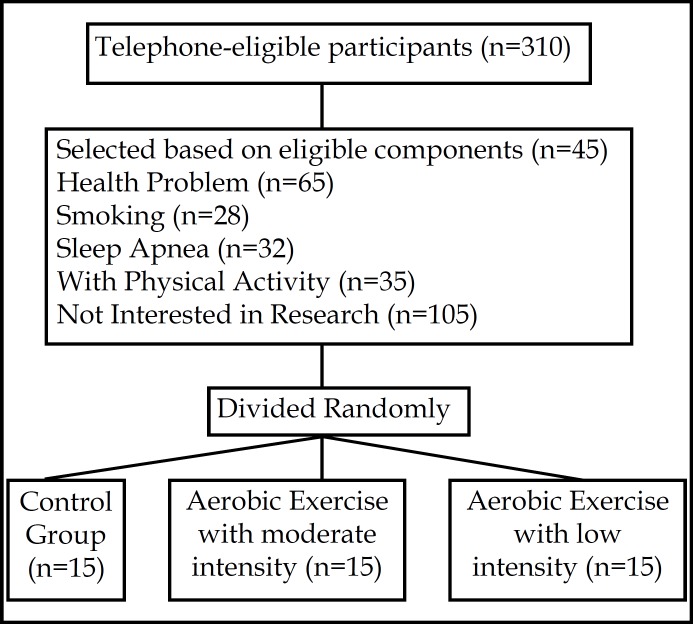
Sampling Procedure

***Participants:*** A total of 45 men older adults with ages between 60 to 70 years participated in this research. Subjects were selected through the eligibility components (Fig.1) and divided randomly in two experimental groups (aerobic exercise with low and moderate intensity) and one control group. In each group selected 15 older adults based on inclusion and exclusion criteria (Figure 1).

***Eligibility Components:*** The eligibility components consisted of (a) age between 60 to 70 years, (b) without sleep apnea, (c) not smoking, (d) not engaged particularly in moderate and vigorous physical activity, (e) no taking hypnotic drugs, (f) without any musculoskeletal problems that would prevent participation in aerobic exercises.

***Interventions:*** For safety and risks associated with aerobic exercises in older adults, all of them were evaluated by a doctor to confirm that their physical and mental health. Also, the maximum heart rate (MaxHR) was obtained by subtracting one's age from 220. Furthermore, based on aerobic exercise type (40-50% MaxHR for low intensity group and 60-70% MaxHR for moderate intensity group) the target MaxHR was calculated for each subject. 

The exercise protocol consisted of 8 weeks aerobic exercises (2 sessions in per week) based on Rockport one-mile walking/running test. Participants in the low intensity (40-50% MaxHR) and moderate intensity (60-70% MaxHR) groups conducted their exercise protocols, and the control group continued their daily activities. Moreover, the exercise intensity was evaluated and controlled with uses specific chest belts. The exercise program consisted of warm-up (10 minutes with 20-30% MaxHR), 35 minutes of low or moderate aerobic exercises and cool-down in 10 minutes, respectively.^13^^,^^17^

Also, all sessions had been done by two examiners who were experts in exercise and work with older adults. Furthermore, this study was conducted according to the Ethical Committee of the University of Social Welfare and Rehabilitation Sciences.

***Measures:*** To measure sleep quality, we used the validated Petersburg Sleep Quality Index (PSQI) in pre-test and post-test stages.^13^^,^^14^ The PSQI is an 18-item questionnaire and assesses sleep quality and disturbances in the recent month. It is a total measure and seven components such as sleep latency, sleep duration, sleep efficacy, sleep disturbance, perceived sleep quality, use of sleep medication and daytime dysfunction.^10^

The collected data have been analyzed with using inferential statistics such as one-way ANOVA and Tokey Post Hoc tests at the P<0.05 significant level with SPSS version 16.

## RESULTS

The descriptive results related to global sleep quality and their components in pre-test and post-test stages are presented in Table-I.

**Table-I:** Global sleep quality and their components scores in pre-test and post-test

**Components**	**Groups**	**Pre-test**	**Post-test**
Perceived Sleep Quality	High intensity Group	0.67±0.13	0.32±0.007
	Low intensity Group	0.73 ±0.12	0.53±0.14
	Control Group	0.77±0.008	0.79±0.06
Sleep Latency	High intensity Group	0.70±0.18	0.35±0.10
	Low intensity Group	0.67±0.15	0.46±0.16
	Control Group	0.68±0.16	0.74±0.17
Sleep Duration	High intensity Group	0.77±0.15	0.39±0.07
	Low intensity Group	0.74±0.16	0.61±0.13
	Control Group	0.80±0.15	0.81±0.14
Sleep Efficacy	High intensity Group	0.71±0.12	0.37±0.09
	Low intensity Group	0.70±0.13	0.53±0.10
	Control Group	0.69±0.11	0.70±0.12
Sleep Disturbance	High intensity Group	0.73±0.15	0.33±0.08
	Low intensity Group	0.69±0.14	0.58±0.11
	Control Group	0.72±0.16	0.71±0.13
Use of Sleep Medication	High intensity Group	0.81±0.11	0.36±0.07
	Low intensity Group	0.79±0.12	0.54±0.09
	Control Group	0.74±0.14	0.72±0.10
Daytime Dysfunction	High intensity Group	0.81±0.22	0.35±0.12
	Low intensity Group	0.74±0.24	0.51±0.10
	Control Group	0.73±0.19	0.72±0.17
Total Sleep Quality	High intensity Group	5.20±1.06	2.47±0.62
	Low intensity Group	5.60±1.07	3.76±0.83
	Control Group	5.12±0.95	5.19±0.89

On the other hand, the pre-test and post-test scores were analyzed with one-way ANOVA test. Results in pre-test stage showed that there are no significant differences between 3 groups in global sleep quality and their components (P>0.05). Also, the results in post-test stage showed that there are significant differences between groups in all variables (P<0.05). Based on one-way ANOVA results, the perceived sleep quality component with F(2,42)=15.570, the sleep latency component with F(2,42)=9.690, the sleep duration component with F(2,42)=16.165, the sleep efficacy component with F(2,42)=11.790, the sleep disturbance component with F(2,42)=12.710, the use of sleep medication component with F(2,42)=15.750, and the daytime dysfunction component with F(2,42)=10.450 were significant at the level of P<0.05. Also, the total sleep quality scores with F(2,42)=110.0760 was significant (P<0.05).

**Table-II:** One-way ANOVA results in post-test stage

*Components*	*Variance*	*Sum of Square*	*Mean of Square*	*Df*	*F*	*P*
Perceived Sleep Quality	Between Group	4.80	2.40	2	**15.750**	0.001*
	Within Group	6.40	0.151	42		
	Total	11.20	------	44		
Sleep Latency	Between Group	4.82	2.42	2	**9.690**	0.001*
	Within Group	10.40	0.152	42		
	Total	15.22	------	44		
Sleep Duration	Between Group	5.64	2.82	2	**16.165**	0.001*
	Within Group	7.33	0.175	42		
	Total	12.97	------	44		
Sleep Efficacy	Between Group	4.04	2.02	2	**11.790**	0.001*
	Within Group	7.20	0.171	42		
	Total	11.24	------	44		
Sleep Disturbance	Between Group	5.64	2.82	2	**12.710**	0.001*
	Within Group	9.33	0.222	42		
	Total	14.91	------	44		
Use of Sleep Medication	Between Group	4.81	2.41	2	**15.750**	0.001*
	Within Group	6.45	0.153	42		
	Total	11.26	------	44		
Daytime Dysfunction	Between Group	5.66	2.83	2	**10.450**	0.001*
	Within Group	11.33	0.270	42		
	Total	16.99	------	44		
Total Sleep Quality	Between Group	246.711	123.356	2	**110.076**	0.001*
	Within Group	47.067	1.122	42		
	Total	293.778	------	44		

* *Significant level at the P<0.05*

Also, the Tukey Post Hoc test showed that the moderate intensity group scores in sleep quality and its components were better than other groups (P<0.05). Based on Tukey Post Hoc test, the high intensity group scores in perceived sleep quality component with P<0.021, in the sleep latency component with P<0.042, in the sleep duration component with P<0.011, in the sleep efficacy component with P<0.035, in the sleep disturbance component with P<0.026, in the sleep medication component with P<0.024, and in the daytime dysfunction component with P<0.045 were significant related to other groups. Finally, the low intensity group scores were better than control group (P<0.05). For more details see Tables I and II.

## DISCUSSION

Sleep disturbance is one of the important problems that are caused by age related changes in human. With increasing age, inappropriate changes are created in sleep structure and sleep cycle. So that, studies have shown that poor sleep quality in older adults is directly associated with physical and mental impairments.^18^^,^^19^ Thus the purpose of this study was to investigate the effect of low and moderate intensity aerobic exercises on sleep quality and its components in men older adults.

Our results from this study indicate that eight-week moderate-intensity aerobic exercise was effective on improving sleep quality and all its components in men older adults. These results highlight the potential of structured field aerobic exercise programs to improve these variables. Our results are in agreement with well-controlled laboratory researches.^11^^,^^13^ Accordingly, King and colleagues in their study found that the moderate-intensity endurance exercise may have modest positive effects on several dimensions of sleep quality aspects. Researchers reported that the 12-month exercise with moderate-intensity shift observed from Stage 1 to Stage 2 sleep and the reduced number of awakenings observed during this sleep phase.^13^ Furthermore, Reid et al. reported that the 6-week program of moderate aerobic exercise plus sleep hygiene education is effective in improving self reported sleep quality, mood and quality of life in older adults with insomnia. In this study, the increase in sleep duration by 1.25 hours was higher than what has been reported for other non-pharmacological interventions for insomnia.^11^

The mechanisms underlying these results could be explained by the thermoregulatory theory.^20^ Murphy & Campbell argued that that sleep onset is the evening decline in body temperature, which is primarily mediated by increased peripheral skin blood flow.^20^ Sleep onset is associated with peripheral heat dissipation through vasodilation and increased sweating, together with a reduction in metabolic rate and core body temperature during sleep.^11^ Furthermore, body temperature is regulated at a lower level during non-REM sleep than during wakefulness while thermoregulation is inhibited during REM sleep.^11^^,^^20^ Also, in this mechanism, the anterior hypothalamus region plays a vital role in sleep and temperature regulation,^20^ so that body temperature elevation before bed-time can activate both heat-loss and the associated sleep mechanisms.^11^^,^^20^ On the other hand, there are two types of the energy conservation theory. One type is that sleep is for the reduction of energy expenditure below the level attainable by rest alone^11^, the second type suggests that the sleep sets a limit on metabolic expenditure to the extent necessary to balance a species’ energy budget.^11^^,^^20^ some evidence related to the energy conservation function for sleep is that energy expenditure is only 10–15 percents less than that associated with quiet wakefulness.^11^

Taken together, we conclude that the field moderate-intensity aerobic exercise has a positive effect on sleep quality and its components. Thus, the field moderate-intensity aerobic exercise is a useful and therapeutic method recommended for older adults. 

## Authors Contributions:

**AAAK:** Designed the protocol and editing of manuscript

**AS, PSD:** Involved in data collection and manuscript writing

**RM: **Involved in data collection
